# Cardioversion of Post-Ablation Atrial Tachyarrhythmia with Ibutilide and Amiodarone: A Registry-Based Cohort Study

**DOI:** 10.3390/ijerph19116606

**Published:** 2022-05-28

**Authors:** Filippo Cacioppo, Michael Schwameis, Nikola Schuetz, Julia Oppenauer, Sebastian Schnaubelt, Alexander Simon, Martin Lutnik, Sophie Gupta, Dominik Roth, Harald Herkner, Alexander Oskar Spiel, Anton Norbert Laggner, Hans Domanovits, Jan Niederdoeckl

**Affiliations:** 1 Medical University of Vienna, Department of Emergency Medicine, Waehringer Guertel 18-20, 1090 Vienna, Austria; filippo.cacioppo@meduniwien.ac.at (F.C.); michael.schwameis@meduniwien.ac.at (M.S.); nikola.schuetz@meduniwien.ac.at (N.S.); julia.oppenauer@meduniwien.ac.at (J.O.); sebastian.schnaubelt@meduniwien.ac.at (S.S.); sophie.gupta@meduniwien.ac.at (S.G.); dominik.roth@meduniwien.ac.at (D.R.); harald.herkner@meduniwien.ac.at (H.H.); anton.laggner@meduniwien.ac.at (A.N.L.); hans.domanovits@meduniwien.ac.at (H.D.); jan.niederdoeckl@meduniwien.ac.at (J.N.); 2 Clinic Ottakring, Department of Emergency Medicine, Montleartstraße 37, 1160 Vienna, Austria; alexander.simon@gesundheitsverbund.at; 3 Medical University of Vienna, Department of Clinical Pharmacology, Waehringer Guertel 18-20, 1090 Vienna, Austria; martin.lutnik@meduniwien.ac.at

**Keywords:** tachycardia, supraventricular, catheter ablation, Amiodarone, Ibutilide

## Abstract

Patients with recurrence of atrial tachyarrhythmia after catheter ablation for atrial fibrillation or atrial flutter constitute a rapidly growing cohort, but study-driven treatment recommendations are lacking. The present study aimed to compare the cardioversion success of ibutilide and amiodarone in patients with post-ablation atrial tachyarrhythmia. We included all episodes of post-ablation atrial tachyarrhythmia in patients treated with either intravenous ibutilide or amiodarone at an academic emergency department from 2010 to 2018. The primary endpoint was the conversion to sinus rhythm. The conversion rates were stratified by arrhythmia type, and multivariable cluster-adjusted logistic regression was used to estimate the effect of ibutilide and amiodarone on cardioversion success, given as the odds ratio (OR) with 95% confidence intervals (95% CI). In total, 109 episodes of 72 patients were analyzed. The conversion rates were 37/49 (76%) for ibutilide and 16/60 (27%) for amiodarone. Compared to amiodarone, ibutilide was associated with higher odds of conversion (multivariable cluster-adjusted OR 5.6, 95% CI 1.3–24.3). The cardioversion success of ibutilide was the highest in atrial flutter (crude OR 19.5, 95% CI 3.4–112.5) and focal atrial tachycardia (crude OR 8.3, 95% CI 1.5–47.2), but it was less pronounced in atrial fibrillation (crude OR 4.5, 95% CI 1.2–17.2). Randomized trials are warranted to confirm our findings.

## 1. Introduction

The current guidelines recommend catheter-based interventions such as pulmonary vein isolation in patients with atrial fibrillation (AF) or cavotricuspid isthmus ablation in patients with typical atrial flutter (AFL) [1,2]. They are a promising and potentially curative approach for the growing number of patients affected by these arrhythmias. Despite rapidly evolving technical and methodological improvements, the recurrence rates after catheter-based interventions in patients with AF are still up to 45% [3,4,5,6,7]. The frequency of recurrence depends on the classification of AF, the technique used and the extent of ablation, among other factors [8,9]. Patients with post-ablation atrial tachyarrhythmias often show unusual ECG rhythms such as focal atrial tachycardia (fAT) or atypical AFL [10]. Electrophysiological studies in catheter laboratories revealed several complex mechanisms leading to these post-ablation atrial tachyarrhythmias. Gaps in ablation lines or reentries around scars as a residual of previous catheter-based procedures are frequently identified as the cause [11,12]. Due to persistent symptoms, patients with post-ablation atrial tachyarrhythmia often seek help not only in specialized centers of care but also in emergency departments (ED). There, in addition to adequate rate control, the restoration of sinus rhythm is often the primary goal. Ibutilide and amiodarone are commonly used in emergency departments to treat AF and AFL. Both are class III antiarrhythmic agents but differ in their pharmacological mechanism of action: Ibutilide prolongs the action potential duration by enhancing a slow, inward sodium current [13], whereas amiodarone is a potassium channel blocker with additional weak calcium and a sodium channel as well as beta-receptor blocking properties [14]. Ibutilide is known to be more effective in the cardioversion of macro-reentry tachycardia such as AFL [15], whereas, in AF, amiodarone and ibutilide seem to be equally effective [16]. However, for patients suffering from recurrent atrial tachyarrhythmias after catheter ablation, there are no evidence-based recommendations for drug treatment. Because of the different pathomechanisms leading to post-ablation atrial tachyarrhythmias, it is unclear whether the conversion rates of amiodarone and ibutilide differ from those of AF and AFL. Therefore, we aimed to analyze the effectiveness of ibutilide and amiodarone in patients with post-ablation atrial tachyarrhythmia.

## 2. Materials and Methods

### 2.1. Study Population

For the present cohort study, all cases of atrial tachyarrhythmia (AF or atrial tachycardia (AFL, fAT)) presented at the Department of Emergency Medicine of the Medical University of Vienna between July 2010 and October 2018 were eligible. We included all consecutive adult patients who had had at least one catheter ablation due to AF or AFL and received either ibutilide or amiodarone during their stay at the ED.

The Department of Emergency Medicine comprises an outpatient care section and an affiliated emergency ward with monitor beds and a seven-bed critical care unit, covering up to 90,000 patients per year overall [17].

The patients were recruited from the department’s arrhythmia registry. Established in 2009, the arrhythmia registry prospectively includes all patients diagnosed with supraventricular tachycardia, as confirmed by 12-lead electrocardiography. Demographics, past medical history including comorbidities, concomitant medication and previous attempts at electrical cardioversion, CHA2DS2-VASc score, the results from blood gas analyses, blood count, blood chemistry, coagulation parameters, thyroid function and troponin-T and N-terminal pro b-type natriuretic peptide (NT-proBNP) levels were obtained. Additionally, vital signs including heart rate, blood pressure and oxygen saturation, symptoms attributable to the arrhythmia, the time of symptom onset, ECG characteristics such as QTc (automatically calculated by the ECG machine using Bazett’s formula) and treatments including electrolyte substitution, rate control medication, cardioversion attempts and the occurrence of adverse events (QTc prolongation, ventricular tachycardia, bradycardia, hypotension) were documented by study fellows. Until October 2018, a total of 3426 episodes were documented, with 515 episodes being a recurrence of atrial tachyarrhythmia after catheter ablation for AF or AFL.

The registry was approved by the local ethics committee (EC number: 1568/2014) and is registered at ClinicalTrials.gov (NCT03272620).

### 2.2. ECG Classification

We reviewed the 12-lead ECGs and divided AF and atrial tachycardia. We further categorized atrial tachycardia into clockwise AFL, counterclockwise AFL, atypical AFL or fAT. The categorization criteria were based on the 2015 ACC/AHA/HRS Guideline for the Management of Adult Patients with Supraventricular Tachycardia definition [18].

### 2.3. Application of Medication

The dosing of ibutilide was performed according to the manufacturer’s recommendations (in patients with ≥60 kg: 1 mg over a 10 min period; the infusion was repeated once if the arrhythmia was still present after a 10 min observation period; in patients with <60 kg, the dosage was reduced to 0.01 mg/kg). The primary dose of amiodarone was 300 mg and was administered intravenously over 20 to 30 min. Whether the amiodarone dosage was further increased was at the discretion of the treating physician.

### 2.4. Statistics

We present the continuous data as the median and interquartile range (IQR) or the mean and standard deviation (SD), as appropriate. The categorical data are shown in absolute counts and relative frequencies.

Logistic regression was used to estimate the effect of ibutilide versus amiodarone on cardioversion success. Based on the existing evidence and clinical considerations, we identified variables associated with risk factors and outcome a priori. Among these, we included age, gender, NT-proBNP-levels, duration of arrhythmia (onset) and ECG-rhythm as covariables in our logistic regression model. To allow for multiple episodes per included patient, we used cluster-robust methods. The results are presented as odds ratios with a 95% confidence interval. For a sensitivity analysis, we used structural equation modelling and compared its results with the conventional logistic regression. We performed subgroup analyses according to the presenting type of arrhythmia, but, owing to the sample size, we used crude estimates and abstained from a formal test for interaction. For data management and analysis, we used MS Excel, IBM SPSS Statistics 25 and Stata 13SE (Stata Corp, College Station, TX, USA).

## 3. Results

**Figure 1:**
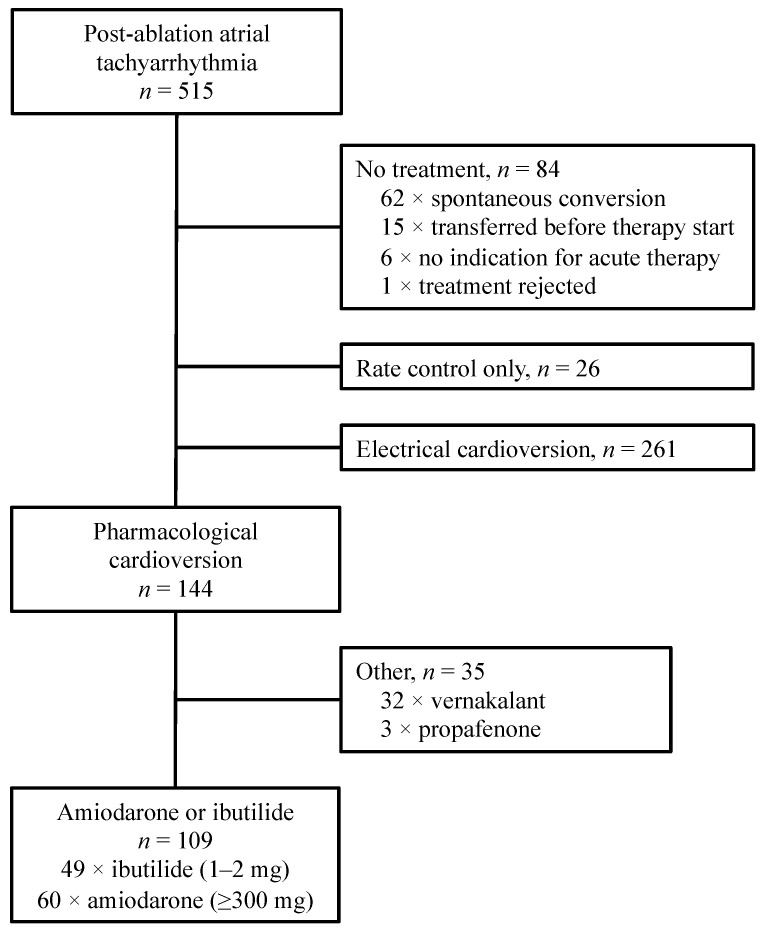
Course of treatment of atrial fibrillation, atrial flutter and focal atrial tachycardia in patients with post-ablation atrial tachyarrhythmia. Patients receiving less than 300 mg of amiodarone were classified as rate control. We did not include vernakalant in our analysis, as it is only approved for patients with atrial fibrillation.

Between July 2010 and October 2018, a total of 515 episodes of post-ablation atrial tachyarrhythmia were treated at the Department of Emergency Medicine of the Medical University of Vienna (Figure 1). Pharmacological cardioversion was attempted in 144 episodes. In 109 episodes, either amiodarone (*n* = 60) or ibutilide (*n* = 49) was administered. There was no episode involving the administration of both amiodarone and ibutilide. The patients’ baseline characteristics are shown in Table 1. Briefly, the patients receiving amiodarone were more likely to be male and showed higher rates of comorbidities such as coronary artery disease or hypertension. There was no remarkable difference in terms of onset time, maximum heart rate in the ECG or laboratory values.

**Figure 2:**
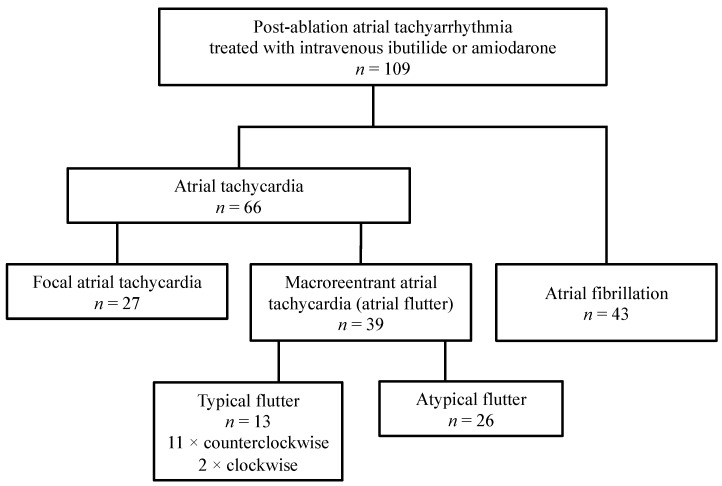
Classification of ECGs and subgroup sizes. Atrial tachycardia (66 episodes in 45 patients) was more common than atrial fibrillation (43 episodes in 37 patients) and was divided into focal atrial tachycardia (27 episodes in 17 patients), typical flutter (13 episodes in 13 patients) and atypical flutter (26 episodes in 16 patients).

**Figure 3:**
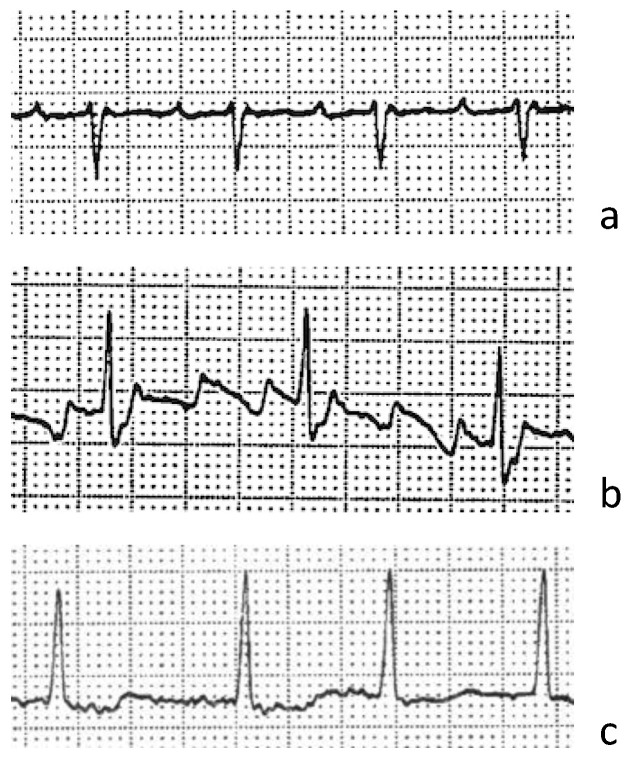
ECG example for (**a**) focal atrial tachycardia, (**b**) atrial flutter and (**c**) atrial fibrillation.

The classification of ECGs and subgroup sizes is shown in Figure 2; ECG examples are shown in Figure 3.

### 3.1. Cardioversion Success

**Figure 4:**
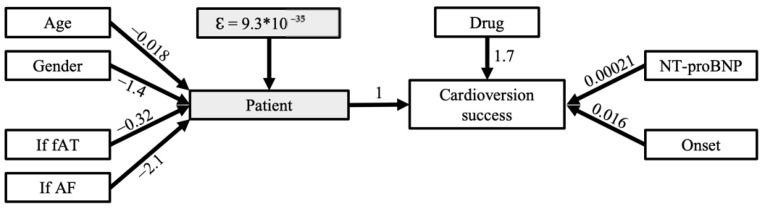
Structural equation modeling. White boxes are measured variables, grey boxes are calculated variables. Cardioversion success is the outcome variable. Numbers above the arrows indicate the influence if the variable changes per one unit. fAT = focal atrial tachycardia, AF = atrial fibrillation.

**Figure 5:**
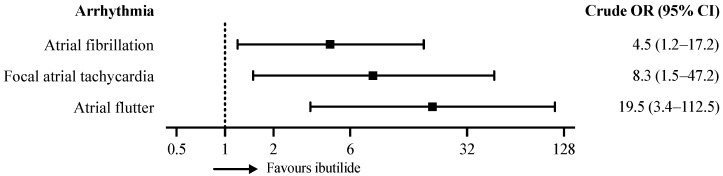
Crude Odds ratios and 95% CI for tachycardia subgroups.

The contingency tables for the total sample and all of the subgroups are presented in Table 2. Overall, the cardioversion success rates were 37/49 (76%) for ibutilide and 16/60 (27%) for amiodarone (crude OR 8.5 (95% CI 3.6–20.2)). Adjusting for the covariables resulted in a multivariable cluster-adjusted OR of 5.6 (95% CI 1.3–24.3). Using structural equation modelling, we were able to describe a latent variable using age, gender and rhythm. This latent variable represents general patient characteristics independent of the current episode. The results of the structural equation modelling were asymptotically identical to the results of the multivariable logistic regression (Figure 4). The treatment effects differed between the three post-ablation tachyarrhythmia subgroups, as depicted in Figure 5. The cardioversion success of ibutilide was most pronounced in AFL (crude OR 19.5, 95% CI 3.4–112.5) and fAT (crude OR 8.3, 95% CI 1.5–47.2).

### 3.2. Dosage and Time to Conversion

In 23 episodes, the conversion to sinus rhythm was achieved after one dose of ibutilide. In the amiodarone group, 50 episodes were treated with a single infusion of 300 mg. Detailed dosing information is presented in Table 3.

The time to conversion after the administration of ibutilide was considerably shorter than that after administration of amiodarone (14 min (IQR 10–31) vs. 72 min (IQR 17–95), respectively). In the episodes that did not convert after drug administration, the therapy attempt was considered unsuccessful after a median time of 135 min (IQR 121–489) in the ibutilide group and 197 min (IQR 90–402) in the amiodarone group.

### 3.3. Adverse Events

In the present cohort, one case of short, self-terminating polymorphic ventricular tachycardia with no hemodynamic deterioration was observed after the administration of ibutilide. In three episodes, a relevant QTc prolongation was found, with a mean prolongation of 53 ms (two after ibutilide, one after amiodarone). In five episodes, the patients showed bradycardia (three after ibutilide, two after amiodarone) but without hemodynamic instability or any other indication for treatment. No other serious adverse events occurred in the analyzed cases.

## 4. Discussion

This analysis is the first to compare the cardioversion success of intravenous ibutilide and amiodarone in patients with post-ablation atrial tachyarrhythmia. Of note, ibutilide showed a substantially higher rate of conversion to sinus rhythm compared to amiodarone, even after adjusting for the relevant covariables. Whereas the difference was most pronounced in patients with AFL and fAT, which were the underlying rhythm disorders in more than half of the analyzed episodes, ibutilide was likewise more effective than amiodarone in patients with AF.

Due to the growing number of catheter ablations in patients with atrial fibrillation and atrial flutter, post-ablation arrhythmias have become a new concern in the ED. Patients with post-ablation arrhythmias often present uncommon types of atrial tachycardia [9]. Depending on the ablation technique used, atrial tachycardia is reported to be responsible for up to 41% of recurrences after catheter ablation [19]. In our study, 61% of the analyzed episodes were atrial tachycardia. However, when analyzing for individual patients, the patients with atrial tachycardia tended to present more repeatedly to the ED (66 episodes in 45 patients, 1.47 episodes/patient) than the patients with AF (43 episodes in 37 patients, 1.16 episodes/patient).

The effectiveness of amiodarone and ibutilide in AF and AFL has been described previously [15,20], but studies conducting a direct comparison of amiodarone and ibutilide are scarce. In 2006, Kafkas et al. published a prospective, randomized trial comparing amiodarone and ibutilide in AF and AFL; however, this did not include information regarding whether some of the studied patients had received catheter ablation before [16]. Their results showed a significantly higher conversion rate of ibutilide in patients with AFL (ibutilide: 87%, amiodarone: 29%, *p* = 0.003) but not in patients with AF (ibutilide: 77%, amiodarone: 69%, *p* = ns).

The conversion rates in our patients with post-ablation AFL were comparable (ibutilide: 90%, amiodarone: 32%) to those in the study by Kafkas et al. However, the conversion rates of post-ablation AF were considerably lower, particularly in patients treated with amiodarone (ibutilide 60%, amiodarone 25%). The difference might be explained by the longer treatment time (continuous infusion of 1200 mg up to 24 h after amiodarone bolus) and observation period (up to 4 h after the cessation of drug therapy) in the study by Kafkas et al., as the conversion time of amiodarone in AF is reported to peak at around 6–8 h after the initiation of therapy [20]. However, a rather short observation period of 2–4 h is more feasible in an ED setting with a rapid patient turnover, and even shorter times defining cardioversion success have been applied in various randomized trials, e.g., 90 min in the AVRO trial [21].

Beyond the effectiveness of ibutilide and amiodarone in AF and AFL after catheter ablation, this study further provides valuable information on the effectiveness of both agents in fAT. In the analyzed episodes of post-ablation fAT, ibutilide was likewise associated with higher conversion rates than amiodarone. In the 2019 ESC Guidelines for the management of patients with supraventricular tachycardia [22], both agents are recommended as class IIb recommendations for patients with fAT. However, this recommendation is based on very limited evidence: Amiodarone is recommended based on a single study published in 1992 analyzing a subgroup of 15 patients with supraventricular tachycardia [23]. In this subgroup, amiodarone was associated with a 100% success rate, but no further details on the arrhythmia mechanisms were presented. For ibutilide, the guidelines cite a single prospective, open-labelled trial evaluating ibutilide in 49 patients with fAT and atypical AFL, which was published in 2006 by Eidher et al. [24]. The overall success rate was 39%, and the subgroup-specific success rates were not reported in detail. In contrast, in our study, ibutilide showed a 71% success rate in fAT and a 100% success rate in atypical AFL. Given that, in our study, all of the included patients had a history of catheter ablation, the crucial pathophysiological difference could be due to this circumstance. In general, the mechanisms of fAT include micro re-entry, triggered activity or automaticity [25]. However, post-ablation fAT is often caused by micro re-entries developing in atrial scars caused by the ablation procedure [26,27]. Due to its prolonging effect on the refractory period, the pharmacological properties of ibutilide appear to be particularly effective in inhibiting these micro re-entries, which may explain its superior effectiveness compared to amiodarone in this subgroup and compared to the episodes analyzed by Eidher et al.

The prolongation of the refractory period after the administration of ibutilide may lead to an increase in the QTc interval, and, in some patients, this even triggers polymorphic ventricular tachycardia. In the previous literature, the frequency of polymorphic ventricular tachycardia after ibutilide administration was about 4% [28]. In our analysis, 1 out of 49 patients (2%) developed polymorphic ventricular tachycardia after the administration of ibutilide. However, the episode was short and self-terminating, and the patient did not show signs of clinical or hemodynamic deterioration. Bradycardia occurred in three patients after the administration of ibutilide (6%) and in two patients after the administration of amiodarone (3%). With amiodarone, bradycardia is a known side effect, and the previously reported rates are comparable to our findings [20]. Bradycardia after ibutilide administration has also been previously reported, but the rates were lower than those in our study [29]. However, our patients had only mild bradycardia and did not require special treatment. It is likely that only more severe forms of bradycardia were considered in previous studies.

Information on the effectiveness of antiarrhythmic drugs in the management of post-ablation atrial tachyarrhythmia is not only important for cardiologists and electrophysiologists but also for emergency physicians, as many of these patients present to EDs. A better understanding of the association between previous catheter ablations, the occurrence of atrial tachycardia and targeted drug use might influence future management. In our study, two main factors may have contributed to the higher conversion rate in patients treated with ibutilide. Firstly, ibutilide is particularly effective in terminating re-entries. As all of the patients had a history of catheter ablation, macro-reentrant tachycardia was common in our population, and, in the patients with post-ablation fAT, micro-reentry was probably a frequent underlying arrhythmia mechanism. Secondly, ibutilide acts faster than amiodarone. Thus, the short patient observation periods in our ED might have further contributed to the relatively low conversion rate of amiodarone.

The particular strength of this study is its real-world-cohort perspective. The study center comprises a 24/7 outpatient clinic and an affiliated inpatient unit, allowing for a fast diagnostic work-up and the enrollment of all treated patients since 2010. However, as this analysis was based on a registry, the treatment approaches and observation periods were not standardized. We therefore used multivariable analyses but cannot rule out residual confounding. Additionally, due to the nature of recurrent tachycardia, some of the patients presented repeatedly to the ED. Since clinicians tend to choose a therapy that has already been proven to be effective, multiple episodes within patients may be correlated. To allow for an interpretation at the patient level, we used cluster robust methods. Further, since patients often present uncommon types of atrial tachycardia after catheter ablation, the differentiation between AF, fAT and AFL (typical vs. atypical) using surface 12-lead ECG remains challenging, even for specialists. To optimize arrhythmia classification, we used the definitions of AF, fAT and AFL provided by international guidelines [18]. In addition, the exact technique used for catheter ablation and the time interval between the procedure and the onset of post-ablation tachyarrhythmias are not documented in our registry, but they may have had an impact on the conversion rates. However, these limitations represent the real-world difficulties of emergency medical decision-making and could thus strengthen the robustness of the results. Finally, the single-center design limits the generalizability of the findings to different settings and populations.

## 5. Conclusions

Ibutilide was associated with higher conversion rates than amiodarone in patients with post-ablation atrial tachyarrhythmia. The association was strongest in the patients with AFL and fAT. As more catheter ablation procedures are performed, the number of patients presenting to the emergency department with post-ablation atrial tachyarrhythmias is likely to increase in the future. Randomized trials are warranted to confirm our findings and provide further insight into the treatment options for these patients.

## Figures and Tables

**Table 1:** Baseline characteristics of the study population. SD = Standard deviation, IQR = interquartile range, CAD = Coronary artery disease, DM = Diabetes mellitus, PAD = Peripheral artery disease, COPD = Chronic obstructive pulmonary disease, DOAC = Direct oral anticoagulant.

	Ibutilide (*n* = 49)	Amiodarone (*n* = 60)
**Clinical characteristics**		
Age, years (SD)	64.16 (12.69)	65.05 (11.45)
Sex, female *n* (%)	29 (59.2%)	27 (45.0%)
Sex, male *n* (%)	20 (40.8%)	33 (55.0%)
Number of ablations, *n* (IQR)	2 (1–3)	2 (1–3)
CHA2DS2-VA^Sc (IQR)	3 (1–3)	2 (1–3)
Max. heart rate ECG, bpm (SD)	131 (16)	131 (26)
Onset, hours (IQR)	5.8 (3.0–15.0)	6.0 (3.0–18.0)
**Laboratory**		
Troponin T, ng/mL (IQR)	0.007 (0.005–0.014)	0.011 (0.009–0.018)
Creatinine, mg/dL (IQR)	0.91 (0.72–1.02)	0.98 (0.87–1.16)
NT-proBNP, ng/L (IQR)	606 (331–1852)	789 (339–1867)
Potassium, mmol/L (SD)	4.10 (0.38)	4.08 (0.40)
**Comorbidities**		
CAD, *n* (%)	2 (4.1%)	8 (13.3%)
Heart failure, *n* (%)	9 (18.4%)	16 (26.7%)
Hypertension, *n* (%)	23 (46.9%)	37 (61.7%)
DM, *n* (%)	4 (8.2%)	5 (8.3%)
Hyperlipidemia, *n* (%)	25 (51.0%)	19 (31.7%)
Hyperthyroidism, *n* (%)	0 (0.0%)	0 (0.0%)
Previous TIA, *n* (%)	1 (2.0%)	1 (1.7%)
Previous stroke, *n* (%)	0 (0.0%)	3 (5.0%)
Previous MCI, *n* (%)	0 (0.0%)	3 (5.0%)
PAD, *n* (%)	1 (2.0%)	0 (0.0%)
COPD, *n* (%)	3 (6.1%)	4 (6.7%)
Current smoker, *n* (%)	1 (2.0%)	1 (1.7%)
Previous smoker, *n* (%)	4 (8.2%)	9 (15.0%)
**Home treatment**		
DOACs, *n* (%)	8 (16.3%)	19 (31.7%)
Phenprocoumon, *n* (%)	17 (34.7%)	22 (36.7%)
Betablockers, *n* (%)	20 (40.8%)	32 (53.3%)
Diuretics, *n* (%)	6 (12.2%)	15 (25.0%)

**Table 2:** Conversion rates and odds ratios for amiodarone and ibutilide in the total population and the subgroups. Multivar. OR = Multivariable cluster-adjusted odds ratio.

Arrhythmia	Ibutilide	Amiodarone	Crude OR (95% CI)	Multivar. OR (95% CI)
Total population (*n* = 109)	37/49 (76%)	16/60 (27%)	8.5 (3.6–20.2)	5.6 (1.3–24.3)
Atrial fibrillation (*n* = 43)	9/15 (60%)	7/28 (25%)	4.5 (1.2–17.2)	
Focal atrial tachycardia (*n* = 27)	10/14 (71%)	3/13 (23%)	8.3 (1.5–47.2)	
Atrial flutter (*n* = 39)	18/20 (90%)	6/19 (32%)	19.5 (3.4–112.5)	
Typical flutter (*n* = 13)	3/5 (60%)	4/8 (50%)		
Atypical flutter (*n* = 26)	15/15 (100%)	2/11 (18%)		

**Table 3:** Dosage of ibutilide and amiodarone. In 25 cases, only one infusion of 1 mg ibutilide was administered: 23 patients converted to sinus rhythm, one patient developed couplets in the ECG and, in one patient, the second dose was not administered due to mild bradycardia. In both cases, electrical cardioversion was successfully performed. In two episodes, a dose of 0.6 mg was used due to a body weight of slightly less than 60 kg. In 21 cases, the second infusion of ibutilide was administered completely; 11 cases subsequently converted to sinus rhythm. In the amiodarone group, a single infusion of 300 mg was used in 50 episodes. In the remaining 10 episodes, a higher dosage was administered.

**Ibutilide**	**1 mg**	**2 mg**	**Other**
Atrial fibrillation	4	10	1 × 1.7 mg
Focal atrial tachycardia	8	6	
Atrial flutter	13	5	2 × 0.6 mg
**Amiodarone**	**300 mg**	**600 mg**	**Other**
Atrial fibrillation	25	2	1 × 330 mg
Focal atrial tachycardia	10	1	1 × 450 mg, 1 × 900 mg
Atrial flutter	15	2	1 × 700 mg, 1 × 680 mg

## Data Availability

The data presented in this study are available on request from the corresponding author.
